# The 256 kb mitochondrial genome of *Clavaria fumosa* is the largest among phylum Basidiomycota and is rich in introns and intronic ORFs

**DOI:** 10.1186/s43008-020-00047-7

**Published:** 2020-11-14

**Authors:** Xu Wang, Yajie Wang, Wen Yao, Jinwen Shen, Mingyue Chen, Ming Gao, Jiening Ren, Qiang Li, Na Liu

**Affiliations:** 1 Present Address: College of Life Sciences, Henan Agricultural University, Zhengzhou, 450002 Henan China; 2 School of Food and Biological Engineering, Chengdu University, Chengdu, 610106 Sichuan China

**Keywords:** Agaricales, Mitochondrial genome, Intron, Evolution, Phylogenetic analysis

## Abstract

In the present study, the complete mitogenome of *Clavaria fumosa*, was sequenced, assembled, and compared. The complete mitogenome of *C. fumosa* is 256,807 bp in length and is the largest mitogenomes among all Basidiomycota mitogenomes reported. Comparative mitogenomic analysis indicated that the *C. fumosa* mitogenome contained the most introns and intronic ORFs among all fungal mitogenomes. Large intergenic regions, intronic regions, accumulation of repeat sequences and plasmid-derived genes together promoted the size expansion of the *C. fumosa* mitogenome. In addition, the *rps3* gene was found subjected to positive selection between some Agaricales species. We found frequent intron gain/loss events in Agaricales mitogenomes, and four novel intron classes were detected in the *C. fumosa* mitogenome. Large-scale gene rearrangements were found occurred in Agaricales species and the *C. fumosa* mitogenome had a unique gene arrangement which differed from other Agaricales species. Phylogenetic analysis for 76 Basidiomycetes based on combined mitochondrial gene sets indicated that mitochondrial genes could be used as effective molecular markers for reconstructing evolution of Basidiomycota. The study served as the first report on the mitogenomes of the family Clavariaceae, which will promote the understanding of the genetics, evolution and taxonomy of *C. fumosa* and related species.

**Supplementary Information:**

**Supplementary information** accompanies this paper at 10.1186/s43008-020-00047-7.

## Introduction

*Clavaria fumosa*, belonging to the family Clavariaceae and the order Agaricales, is a mushroom-forming fungus with dark basidiomata (Kautmanova et al. 2012b). Species of the family Clavariaceae produce simple clubs, coralloid, lamellate-stipitate, hydnoid and resupinate sporocarps (Birkebak et al. 2013). Morphological classification of Clavariaceae species was mostly based on spore characters and presence or absence of clamps (Kautmanova et al. 2012a, b). Compared with other species of Agaricales, Clavariaceae species have few morphological characteristics for taxonomy, which made it difficult to classify or identify species or subspecies from the family Clavariaceae accurately. The introduction of molecular markers, including LSU nrDNA, RNA polymerase II second largest subunit (*rpb2*), nuclear ribosomal 28S, and internal transcribed spacer region (ITS) has promoted the revision of taxonomic concept and phylogeny of Clavariaceae species (Birkebak et al. 2016; Dentinger and McLaughlin 2006; Kautmanova et al. 2012a; Olariaga et al. 2015).

Mitogenome was believed acquired from alphaproteobacteria by eukaryotic ancestors through endosymbiosis (Lang et al. 1999; Munoz-Gomez et al. 2017). In the long-term evolution, mitogenome has been used as a molecular marker to reflect the phylogeny and evolutionary status of fungi (Cameron 2014; Carpi et al. 2016; Fourie et al. 2018; Li et al. 2018c). In addition, the genome organization, content, structure, tRNA gene, repeat sequence, gene arrangement, and also the intron information provided abundant information for understanding the origin and evolution of fungi (Adams et al. 2002; Aguileta et al. 2014; Beaudet et al. 2013; Wang et al. 2020). It was reported that the mitogenome of fungi had a moderate evolution rate, which was between plants (the lowest) and animals (the highest) (Aguileta et al. 2014). Despite large variations in mitochondrial content (Li et al. 2019e; Sandor et al. 2018), most fungal species contained a set of core protein coding genes (PCGs), which included *atp6*, *atp8*, *atp9*, *cob*, *cox1*, *cox2*, *cox3*, *nad1*, *nad2*, *nad3*, *nad4*, *nad4L*, *nad5*, *nad6*, and *rps3* (Li et al. 2018b, 2019b). The mitogenome of fungi contains various types of introns. As mobile genetic elements in mitogenome, introns could significantly alter the organization and size of fungal mitogenomes. Introns in fungal mitogenome could be divided into different position classes (Pcls) based on their precise insertion position in the coding region of host genes (Férandon et al. 2010). Homologous introns usually belong to the same Pcl and have high sequence similarities. Introns belonging to different Pcls are considered non-homologous and have low sequence similarities. Compared with millions of Basidiomycota species in nature, the number of available Basidiomycota mitogenomes is very limited (less than 120 Basidiomycota mitogenomes available in national center for biotechnology information database), even far less than its counterparts in the animal kingdom. The few mitogenomes available limited our comprehensive understanding of the origin and evolution of Basidiomycota. With the rapid development of the next generation sequencing (NGS) technology, more and more fungal mitogenomes obtained (Li et al. 2020a), which promotes further development of fungal taxonomy and phylogeny.

In the present study, the mitogenome of species from the family Clavariaceae, *C. fumosa*, was sequenced and assembled. The aims of this study are: 1) to reveal the features of the *C. fumosa* mitogenome; 2) to reveal the similarities or variations between *C. fumosa* and other Agaricales mitogenomes in genome size, gene content and gene arrangement by comparative mitogenomic analysis; 3) to reveal the dynamics of introns in Agaricales mitogenomes; 4) to reveal the phylogenetic position of *C. fumosa* in the phylum Basidiomycota based on the combined mitochondrial gene set. This is the first report on the mitogenome from the family Clavariaceae, which will promote the understanding of the origin, evolution and taxonomy of Clavariaceae species and other related species.

## Materials and methods

### Mitogenome sequencing, assembly and annotations

The fruiting bodies of *C. fumosa* were obtained from the Collection Center of Chengdu University (NO. SLZ096). We used a Fungal DNA Kit D3390–00 (Omega Bio-Tek, Norcross, GA, USA) to extract total genomic DNA of the *C. fumosa* fruiting bodies. A NEB Next Ultra II DNA Library Prep Kit (NEB, Beijing, China) was used to construct sequencing libraries with insert size of 200 bp and read length of 150 bp. Whole genomic sequencing (WGS) of *C. fumosa* was performed using an Illumina HiSeq 2500 Platform (Illumina, San Diego, CA, USA). Approximate 20 Gb of raw data was obtained through WGS, and then a series of quality control steps were performed to generate clean reads from the raw sequencing data, which included removing adapter reads by the AdapterRemoval v 2 (Schubert et al. 2016), and filtering low-quality sequences using our own compiling pipeline. The SPAdes 3.9.0 software (Bankevich et al. 2012) was used to assemble the mitogenome of *C. fumosa* using the clean reads we obtained. The MITObim V1.9 (Hahn et al. 2013) software was used to fill gaps between contigs we obtained in previous steps. The obtained complete mitogenome of *C. fumosa* was annotated according to our previously described methods (Li et al. 2018d, 2020b). Briefly, we initially annotated the protein-coding genes (PCGs), rRNA genes, tRNA genes and introns of the *C. fumosa* mitogenome using MFannot (Valach et al. 2014) and MITOS (Bernt et al. 2013), both based on the genetic code 4. PCGs were then modified or predicted using the NCBI Open Reading Frame Finder (https://www.ncbi.nlm.nih.gov/orffinder), and further annotated by BLASTP searches against the NCBI non-redundant protein sequence database (Bleasby and Wootton 1990). The exonerate v2.2 (Slater and Birney 2005) software was used to verify intron-exon borders of PCGs. tRNA genes in the *C. fumosa* mitogenome were also predicted with tRNAscan-SE v1.3.1 (Lowe and Chan 2016). Graphical map of the mitogenome of *C. fumosa* was drawn with OGDraw v1.2 (Lohse et al. 2013).

### Sequence analysis of the *C. fumosa* mitogenome

The DNASTAR Lasergene v7.1 (http://www.dnastar.com/) was used to calculate base compositions of the *C. fumosa* mitogenome and other Agaricales mitogenomes. Strand asymmetries of the Agaricales mitogenomes tested were calculated based on the following formulas: AT skew = [A - T] / [A + T], and GC skew = [G - C] / [G + C]. Sequence Manipulation Suite (Stothard 2000) was used to analyze codon usage within the *C. fumosa* mitogenome, based on the genetic code 4. The nonsynonymous substitution rates (Ka) and synonymous substitution rates (Ks) for core PCGs in the *C. fumosa* mitogenome and other Agaricales mitogenomes reported were calculated using the DnaSP v6.10.01 (Rozas et al. 2017). The genetic distances between each pair of the 15 core PCGs were calculated using MEGA v6.06 (Caspermeyer 2016) based on the Kimura-2-parameter (K2P) substitution model.

### Repetitive element analysis

We conducted BLASTN searches (Chen et al. 2015) of the *C. fumosa* mitogenome against itself to identify if there were intra-genomic duplications of large fragments or interspersed repeats throughout the mitogenome, using an E-value of < 10^− 10^ as a threshold. Tandem repeats (> 10 bp) within the *C. fumosa* mitogenome were detected using the Tandem Repeats Finder (Benson 1999) with default parameters. Repeated sequences in the mitogenome of *C. fumosa* were also detected by REPuter to identify forward (direct), reverse, complemented, and palindromic (revere complemented) repeats (Kurtz et al. 2001). To identify whether there was natural gene segments transferred between the *C. fumosa* mitogenome and its nuclear genome, we conducted BLASTN searches of the mitogenome against the published nuclear genome (CVRD00000000.1).

### Comparative mitogenomic analysis and intron analysis

Comparative mitogenomic analysis was conducted to assess conservation and variations between the reported Agaricales mitogenomes in mitogenome sizes, GC content, base composition, gene arrangement, start and stop codon, gene and intron numbers. Introns of *cox1* genes of the 22 Agaricales mitogenomes were classified into different position classes (Pcls) according to the method described by Férandon et al. (2010). Each Pcl was constituted by introns inserted at the same position in the coding region of the *cox1* gene. The same Pcl from different species usually has a high sequence similarity and contains orthologous intronic ORF.

### Phylogenetic analysis

In order to investigate the phylogenetic position of the *C. fumosa* mitogenome in the phylum Basidiomycota, we constructed a phylogenetic tree of 76 Basidiomycota species based on the combined mitochondrial gene set (15 core PCGs + 2 rRNA genes) according to our previously described methods (Li et al. 2018d). *Annulohypoxylon stygium* (Deng et al. 2018), from the Ascomycota phylum, was used as the outgroup. Individual mitochondrial gene was first aligned using the MAFFT v7.037 software (Katoh et al. 2017). Then the SequenceMatrix v1.7.8 (Vaidya et al. 2011) software was used to concatenate the aligned mitochondrial genes into a combined mitochondrial gene set. A preliminary partition homogeneity test was conducted to detect potential phylogenetic conflicts between different genes. PartitionFinder 2.1.1 (Lanfear et al. 2017) was used to determine best-fit models of evolution and partitioning schemes for the mitochondrial gene set. Both bayesian inference (BI) and maximum likelihood (ML) methods were used to construct the phylogenetic tree. MrBayes v3.2.6 (Ronquist et al. 2012) was used for the BI analysis, and RAxML v 8.0.0 (Stamatakis 2014) was used to perform the ML analysis .

### Data availability

The complete mitogenome of *C. fumosa* was deposited in the GenBank database under the accession number MT114157.

## Results

### Characterization and PCGs of the *C. fumosa* mitogenome

**Fig. 1:**
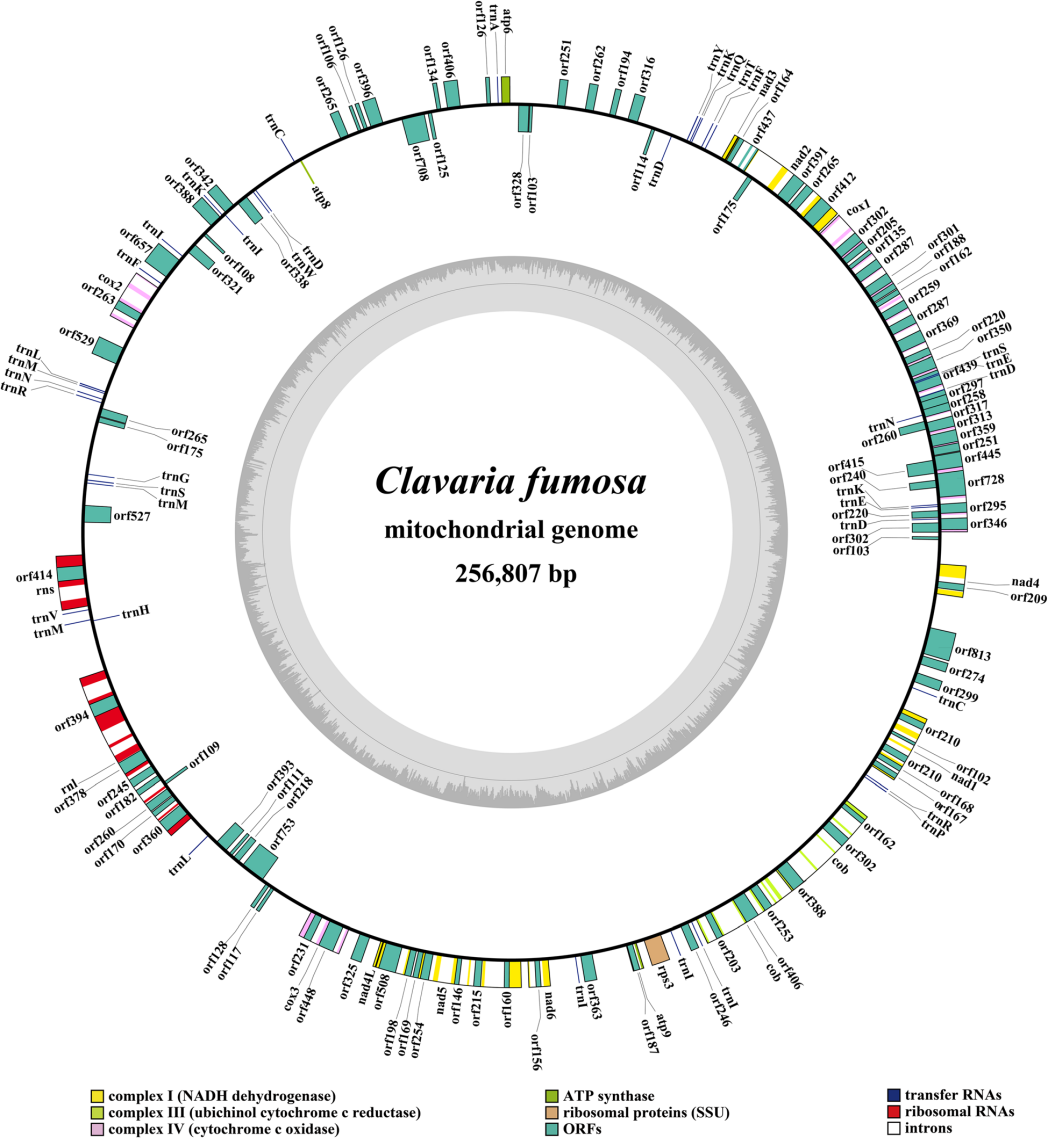
Circular map of the mitochondrial genome of *Clavaria fumosa*. Genes are represented by different colored blocks. Colored blocks outside each ring indicate that the genes are on the direct strand, while colored blocks within the ring indicates that the genes are located on the reverse strand

The mitogenome of *C. fumosa* was composed of circular DNA molecules with a size of 256,807 bp (Fig. 1). The GC content of the *C. fumosa* mitogenome was 19.74%. Both the AT skew and GC skew of the *C. fumosa* mitogenome were positive (Table S1). A total of 121 PCGs were detected in the *C. fumosa* mitogenome, including 46 non-intronic PCGs and 60 intronic ORFs. These 46 non-intronic PCGs contained 15 core PCGs and 31 non-conserved PCGs (Table S2). Non-conserved PCGs in the *C. fumosa* mitogenome included 8 DNA polymerases, 8 RNA polymerases, and 15 proteins with unknown functions. A total of 64 introns were detected in the *C. fumosa* mitogenome, 56 of which belonged to the group I, 2 belonged to the group II, with the remaining 6 introns containing unknown types. The intronic ORFs in the *C. fumosa* mitogenome mainly encoded GIY-YIG endonucleases and LAGLIDADG endonucleases.

### RNA genes and mitogenome compositions

We detected two rRNA genes in the mitogenome of *C. fumosa*, including the small subunit ribosomal RNA (*rns*), and the large subunit ribosomal RNA (*rnl*) (Table S2). Thirty-eight tRNA genes were detected in the mitogenome of *C. fumosa*, which were folded into classical cloverleaf structures (Fig. S1). The *C. fumosa* mitogenome contained 2 tRNAs with different anticodons coding for cysteine, phenylalanine, serine, arginine, leucine, 4 tRNAs with different anticodons coding for Aspartate, and 5 tRNAs with different anticodons coding for Isoleucine. The mitogenome of *C. fumosa* also contained 2 tRNAs with the same anticodons coding for glutamate and asparagine, and 3 tRNAs with the same anticodons coding for lysine and methionine. The length of individual tRNA gene ranged from 70 bp to 86 bp, which was mainly due to variations of the extra arm. Of all the 161 genes detected, 125 genes were located on the direct strand and 36 genes were on the reverse strand.

**Fig. 2:**
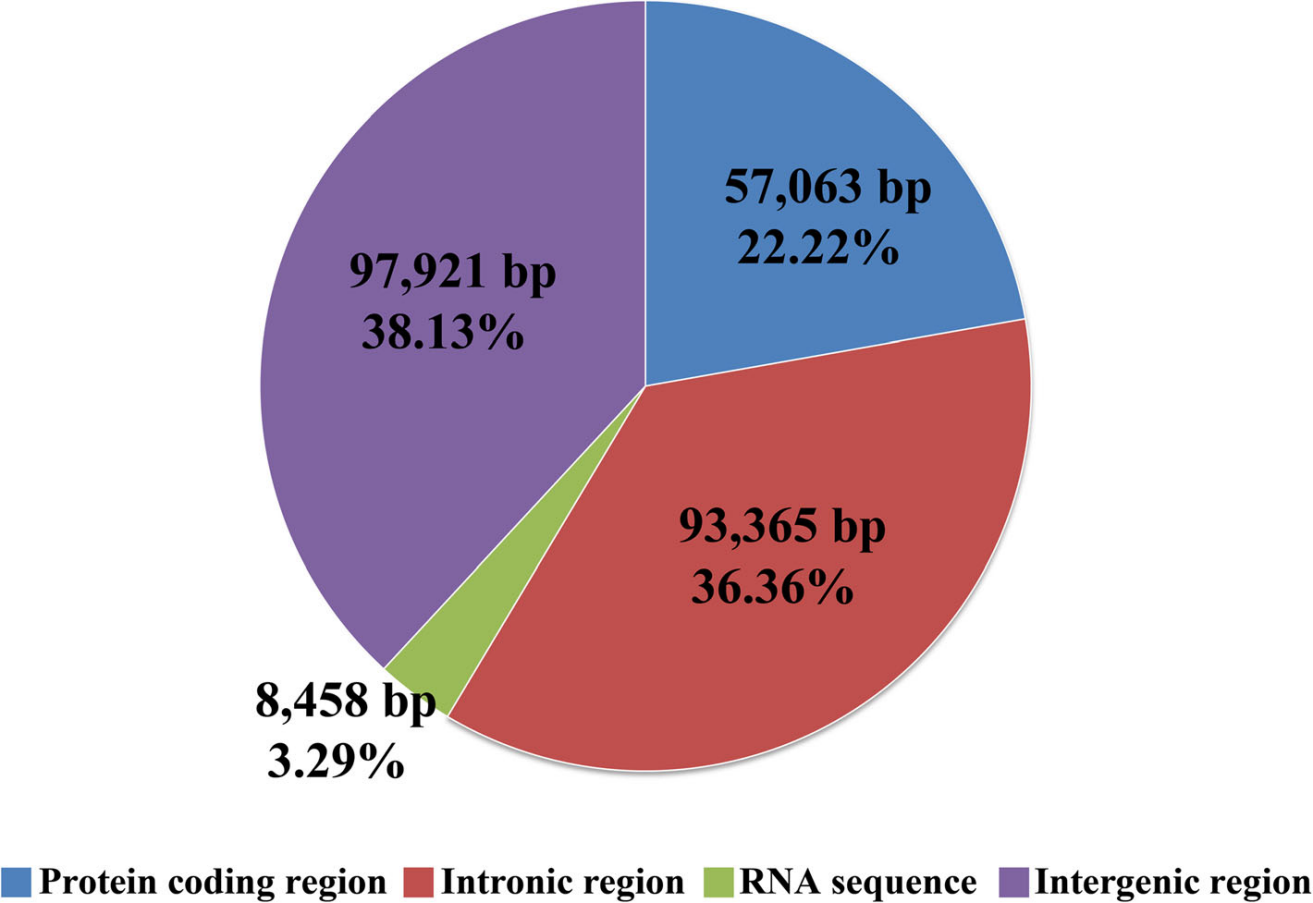
The protein-coding, intronic, intergenic, and RNA gene region proportions of the whole mitochondrial genome of *Clavaria fumosa*

Intergenic region accounted for the largest proportion of the *C. fumosa* mitogenome, comprising 38.13% of the total mitogenome, indicating that the *C. fumosa* mitogenome had a loose structure (Fig. 2). A total of 93,365 bp of intronic sequences were detected in the *C. fumosa* mitogenome, which was the second largest part (36.36%) of the *C. fumosa* mitogenome. Protein coding regions accounted for 22.22% of the *C. fumosa* mitogenome and RNA coding region for 3.29%.

### Codon usage analysis

ATG was the most commonly used start codon in the core PCGs of the 22 *Agricales* species tested, except in the *cox1, nad1* and *nad6* genes, which also used GTG and TTG as start codons (Table S3). TAA was most commonly used as stop codon in core PCGs of the 22 Agaricales species, followed by TAG. Most of the core PCGs in the *C. fumosa* mitogenome used ATG as the start codon, with an exception of the *nad6* gene. The *atp9* and *nad1* genes of the *C. fumosa* mitogenome used TAG as stop codons.

**Fig. 3:**
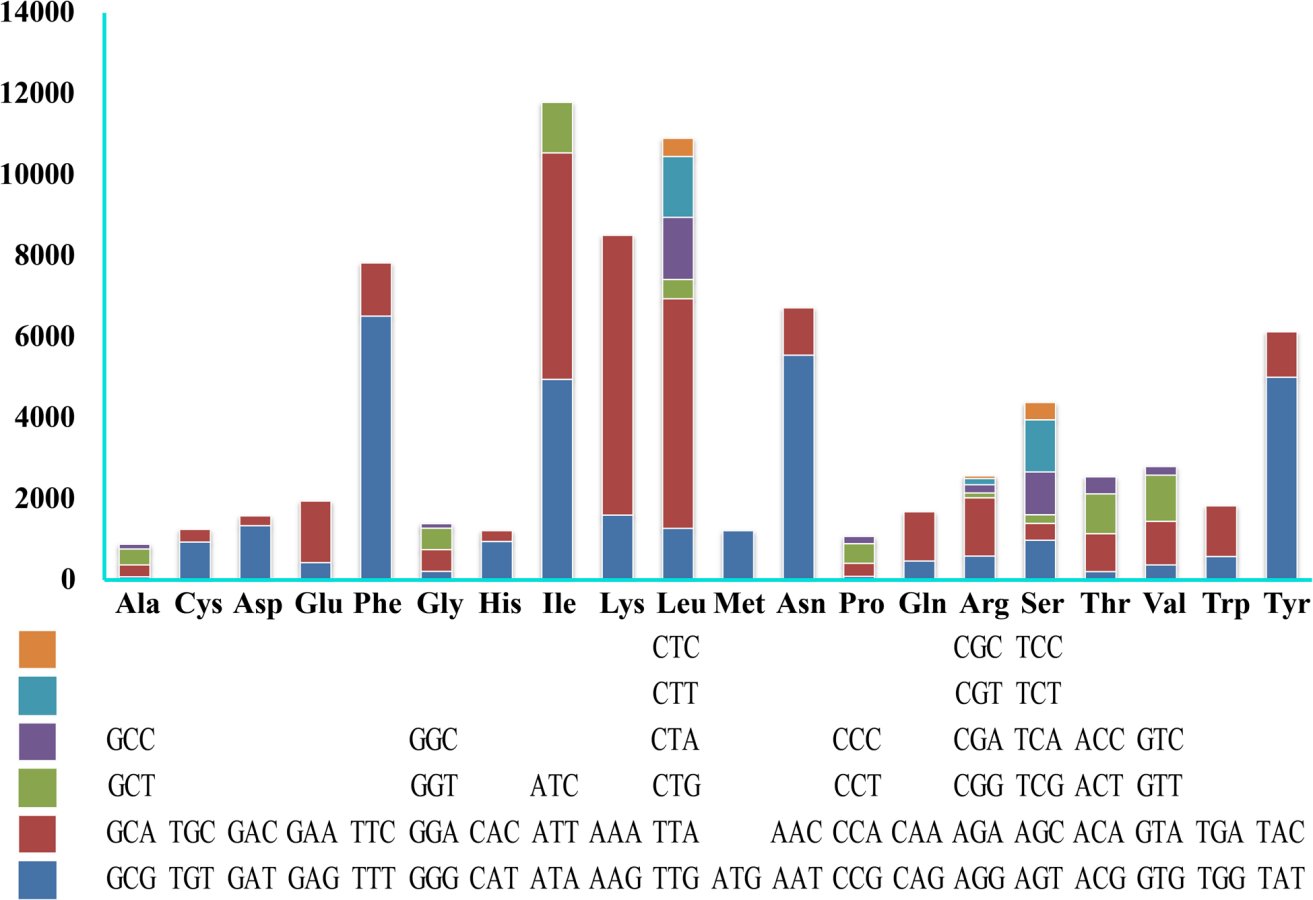
Codon usage in the mitochondrial genome of *Clavaria fumosa*. Frequency of codon usage is plotted on the y-axis

Codon usage analysis indicated that the most frequently used codons in the mitogenome of *C. fumosa* were AAA (for lysine; Lys), TTT (for phenylalanine; Phe), TTA (for leucine; Leu), ATT (for isoleucine; Ile) and AAT (for asparagine; Asn) (Fig. 3). The high frequency of A and T used in codons contributed to the high AT content of the *C. fumosa* mitogenome (80.26%).

### Repeat elements in the *C. fumosa* mitogenome

A total of 178 repetitive sequences were detected in the mitogenome of *C. fumosa* through BLASTN searches of the mitogenome against itself (Table S4). The size of these repetitive sequences ranged from 42 bp to 1302 bp, with nucleotide similarities ranging from 71.22 to 100%. The longest repetitive sequences were located in the first intron of the *nad6* gene, and also in the third intron of the *cox2* gene. A total of 98,181 bp repetitive sequences were detected in the *C. fumosa* mitogenome, which accounted for 38.23% of the whole mitogenome.

A total of 233 tandem repeats were detected in the mitogenome of *C. fumosa*, which accounted for 4.64% of the whole mitogenome (Table S5). The longest tandem sequence in the *C. fumosa* mitogenome was 207 bp, which was located in the intergenic region between neighboring genes *trnM* and *orf517*. About 78.97% tandem repeat sequences in the mitogenome of *C. fumosa* were repeated 2–5 times, and the highest copy number detected was 90 in the *C. fumosa* mitogenome. We also identified 21 forward, 4 palindromic and 25 reverse repeats in the *C. fumosa* mitogenome though REPuter (Table S6), which accounted for 2.61% of the whole mitogenome.

We also conducted BlastN searches of the mitogenome of *C. fumosa* against its nuclear genome to detect if there was any gene fragment that naturally transferred between the mitochondrial and nuclear genomes. A total of 471 aligned fragments were detected between the mitochondrial and nuclear genomes of *C. fumosa* (Table S7). The aligned fragments ranged from 70 bp to 1154 bp and the nucleotide similarities of the aligned fragments ranged from 72.92 to 100%. The largest aligned fragment was located in the first intron region and the second exon region of the *nad2* gene. The second largest aligned fragment was found located in the sixth intron of the *rnl* gene, with a length of 1057 bp. A total of 112,991 bp aligned fragments were detected in the *C. fumosa* mitogenomes, which indicated that natural gene fragment transfer events may have occurred between mitochondrial and nuclear genomes of *C. fumosa*.

### Genetic distance and evolutionary rates of core PCGs

**Fig. 4:**
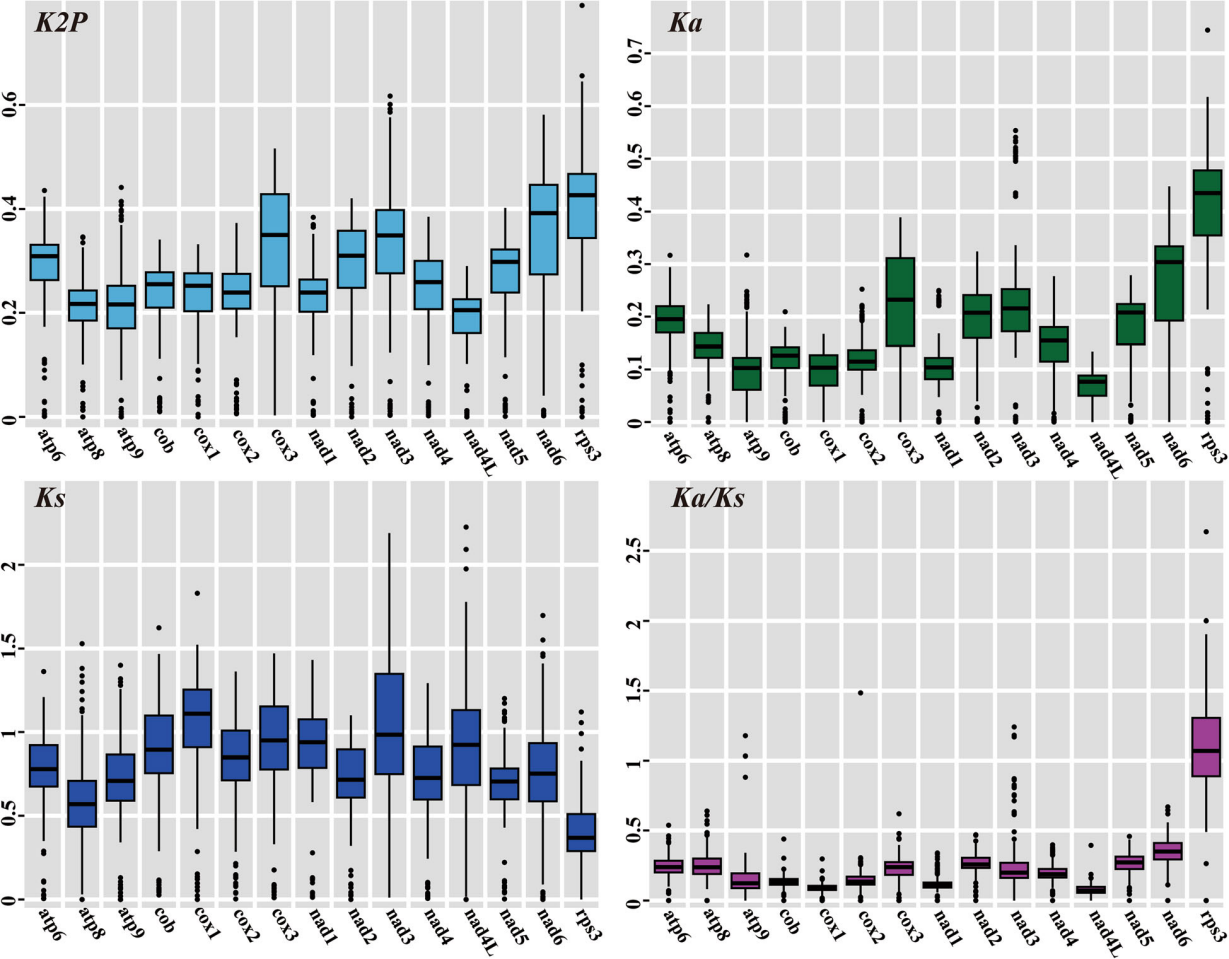
Genetic analysis of 15 core protein coding genes conserved in the 22 Agaricales mitogenomes. K2P, pairwise genetic distances between each pair of the 15 core PCGs in the 22 Agaricales mitogenomes based on the Kimura-2-parameter model; *Ka*, nonsynonymous substitution site; *Ks*, synonymous substitution site. Species and NCBI accession number used for gene arrangement analysis in the present study are listed in Supplementary Table S1

In the present study, we calculated the genetic distance and substitution rates between 22 Agaricales speices reported. The mean K2P genetic distance of the *rps3* gene was the highest among the 15 core PCGs we detected, followed by the *nad6* gene, which showed that the two genes differentiated largely between Agaricales species in the evolutionary process (Fig. 4). The lowest mean genetic distance was observed in the *nad4L* gene, indicating this gene highly conservative between the 22 Agaricales species. The highest non-synonymous substitution rate (*Ka*) was detected in the *rps3* gene, followed by in the *nad6* gene, among the 15 core PCGs we detected. The *nad4L* gene was found exhibited the lowest *Ka* value among the 15 core PCGs detected. The *cox1* gene had the highest synonymous substitution (*Ks*) rate, while the *rps3* gene was observed containing the lowest *Ks* value among the 15 PCGs. The *Ka/Ks* values for 13 out of the 15 core PCGs were less than 1, indicating that these genes were subjected to purifying selection. However, we observed the *Ka/Ks* values of the *nad3* gene were more than 1 between some species, including between *Pleurotus platypus* (Li et al. 2018a) and *Flammulina velutipes* (Yoon et al. 2012), between *Pleurotus eryngii* (Yang et al. 2016) and *Flammulina velutipes*, as well as between *Pleurotus ostreatus* (Wang et al. 2008) and *Flammulina velutipes*. The *Ka/Ks* value of the *rps3* gene was more than 1 between most of the 22 Agaricales species we detected.

### Intron dynamics of *cox1* gene in Agaricales

In this study, we detected 342 introns in the 22 Agricales species, with each species containing 0 to 64 introns (Table S1). Large variations of intron number in Agaricales species indicated that intron gain/loss events have occurred in the evolution of Agaricales species. The *cox1* gene was found to be the largest host gene of Agaricales species, containing 41.52% of the total introns. So in this study, we focused on the dynamic changes of *cox1* gene in Agaricales.

**Fig. 5:**
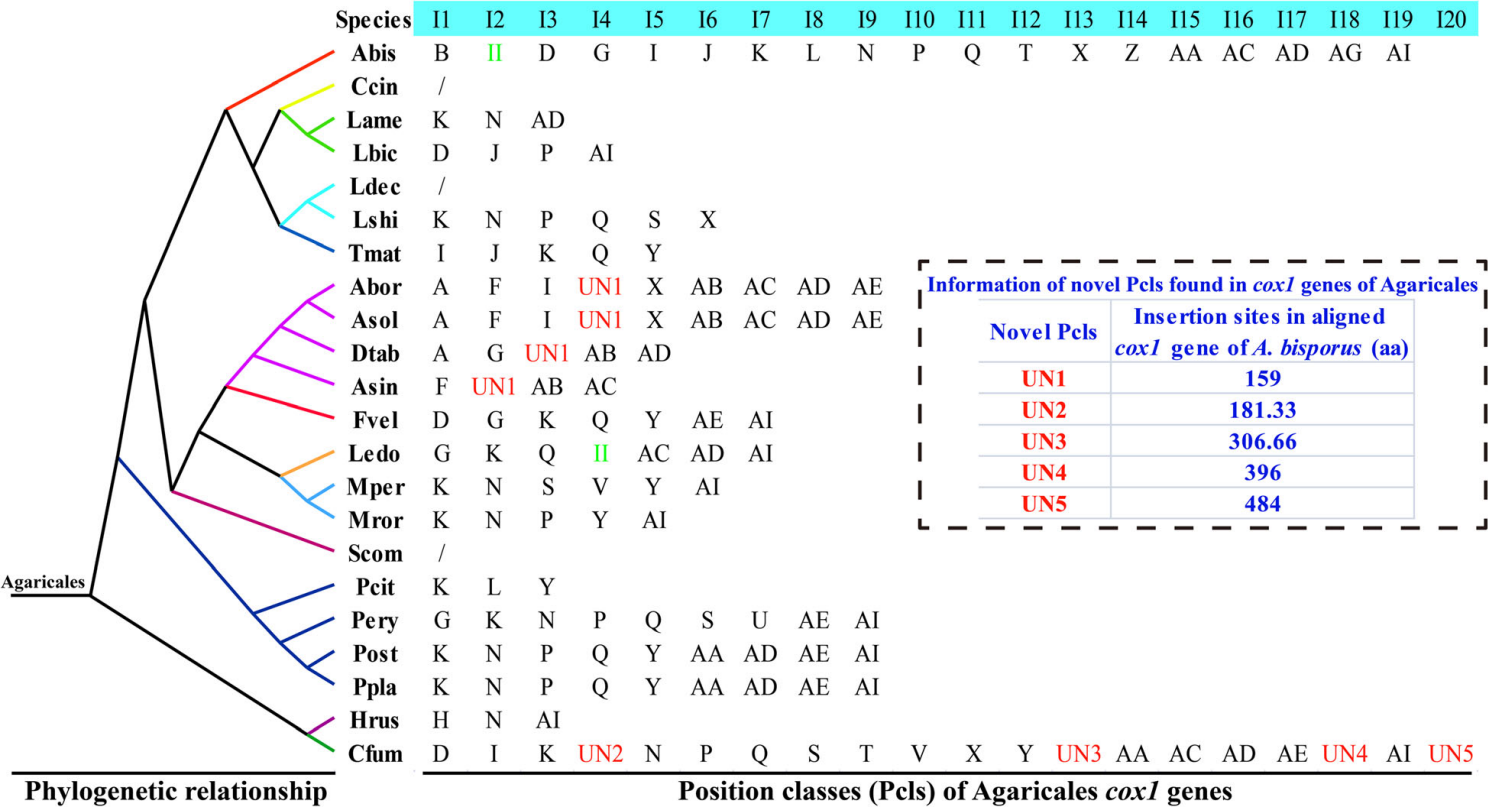
Position class (Pcl) information of *cox1* gene in the 22 Agaricales species. Introns of *cox1* genes of the 22 Agaricales mitogenomes were classified into different position classes (Pcls) according to the method described by Férandon et al. (2010). II represents group II introns, and UN represents novel introns never detected in previous studies. The phylogenetic positions of 22 Agaricales species were established using the Bayesian inference (BI) method and Maximum Likelihood (ML) method based on concatenated mitochondrial genes. Species information is shown in Supplementary Table S1

Of the 142 introns detected in *cox1* gene, 140 belonged to the group I and the other 2 belonged to the group II (Fig. 5). Group I introns in *cox1* genes of the 22 Agaricales species were classified into different Pcls and named alphabetically according to the method described by Férandon et al. (2010). A total of 32 Pcls were detected in Agaricales group I introns, including 5 novel Pcls, which were not reported before. Pcl K was the most widely distributed Pcl in Agaricales, which was distributed in 13 of the 22 Agaricales species. Followed by the Pcl AI, it could be detected in 11 of the 22 Agaricales mitogenomes. However, some Pcls were considered as rare Pcls in Agaricales, including Pcls B, H, U, Z, and AG, which were only distributed in one of the 22 Agaricales species. However, these rare Pcls were detected in mitogenomes of distant species from other taxons, such as *Rhizopogon vinicolor* (Li et al. 2019a) and *Rhizophydium* sp. 136 (Férandon et al. 2010), indicating possible horizontal gene transfer occurred in evolution. The mitogenome of *C. fumosa* contained 4 novel Pcls never detected before, indicating the *C. fumosa* mitogenome contained high diversity of introns. Further studies are needed to reveal the origin and evolution of these novel introns in the *C. fumosa* mitogenome.

### Comparative mitogenome and gene arrangement analyses

Comparative mitogenome analysis indicated that the 256,807 bp mitogenome of *C. fumosa* was the largest among the 22 Agaricales mitogenomes detected (Table S1). The size of the *C. fumosa* mitogenome was 1.90–6.05 times larger than that of other Agaricales species, indicating that the *C. fumosa* mitogenome has experienced a huge size expansion in evolution. The GC content of the *C. fumosa* mitogenome was the third lowest among all Agaricales species detected, which was only higher than *Flammulina velutipes* (16.54%) (Yoon et al. 2012) and *Hygrophorus russula* (19.13%) (Li et al. 2019d), and far lower than the average GC content of Agaricales (26.00%). Among the 22 Agaricales species we detected, 12 species had positive AT skews, while the remaining 10 species contained negative AT skews. Nineteen out of the 22 Agaricales species had positive GC skews. Each Agaricales species contained 15–121 PCGs, and the *C. fumosa* mitogenome had the most PCGs (121), including the most number of intronic ORFs (60) in Agaricales. The *C. fumosa* also contained the largest number of introns, followed by *Agaricus bisporus* (46) (Férandon et al. 2010), and they were also the two species with the largest mitogenome size in Agaricales. All the Agaricales species contained two rRNA genes. In addition, 24–38 tRNA genes were detected in the 22 Agaricales species.

**Fig. 6:**
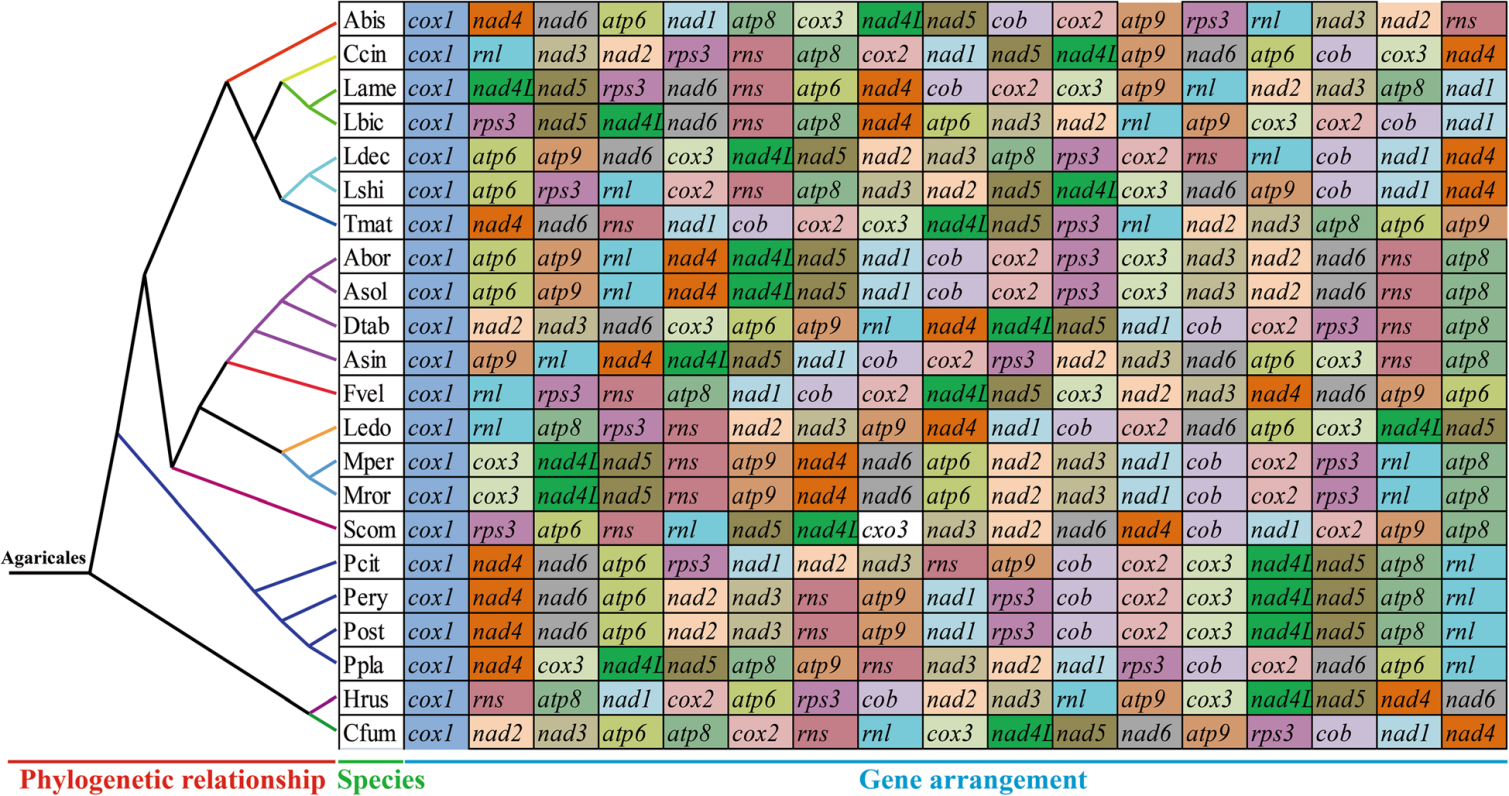
Mitochondrial gene arrangement analyses of 22 Agaricales species. All genes are shown in order of occurrence in the mitochondrial genome, starting from *cox1*. Fifteen core protein coding genes and two rRNA genes were included in the gene arrangement analysis. The phylogenetic positions of 22 Agaricales species were established using the Bayesian inference (BI) method and Maximum Likelihood (ML) method based on concatenated mitochondrial genes. Species and NCBI accession number used for gene arrangement analysis in the present study are listed in Supplementary Table S1

The arrangements of 15 core PCGs and 2 rRNA genes from 22 Agaricales species were detected in this study. We found that the gene arrangement of Agaricales species varied greatly (Fig. 6). Large-scale gene rearrangements were also observed between species from the same genus, including *Laccaria* (Li et al. 2020c), *Lyophylum* (Li et al. 2019c) and *Armilaria* (Kolesnikova et al. 2019). Identical gene arrangements were only observed between two species from the *Moniliophthora* genus (Costa et al. 2012; Formighieri et al. 2008) and between *Pleurotus eringii* and *P. ostratus* from the *Pleurotus* genus (Li et al. 2018a).

### Phylogenetic analysis

**Fig. 7:**
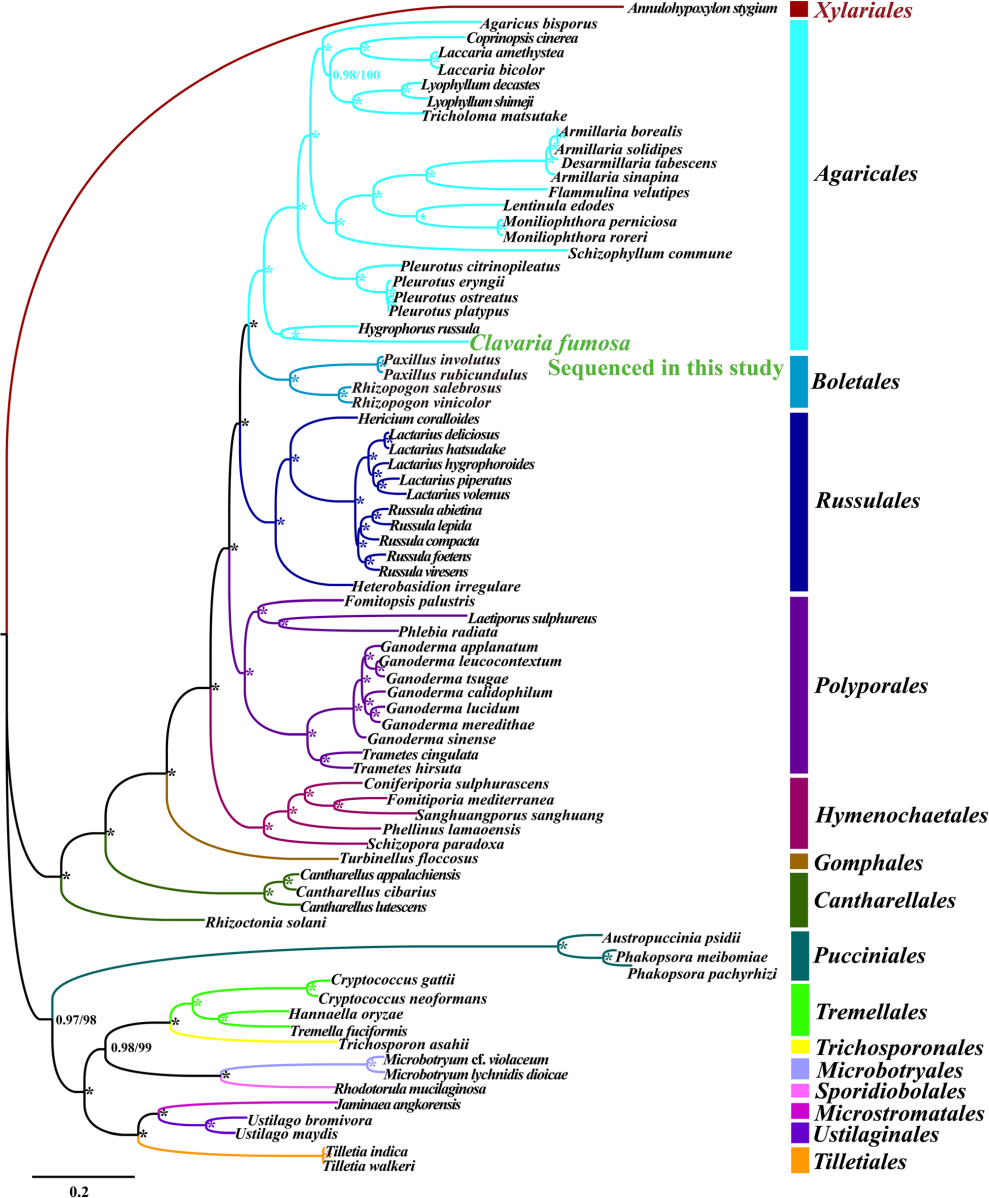
Molecular phylogeny of 76 Basidiomycota species based on Bayesian inference (BI) and Maximum likelihood (ML) analysis of 15 protein coding genes and two rRNA genes. Support values are Bayesian posterior probabilities (before slash) and bootstrap (BS) values (after slash). The asterisk indicates that the BPP value is 1 and the BS value is 100 of the branch. Species and NCBI accession numbers for mitogenomes used in the phylogenetic analysis are provided in Supplementary Table S8

An identical and well-supported phylogenetic tree for 76 Basidiomycota species was obtained using both Bayesian inference (BI) and Maximum likelihood (ML) methods based on the combined mitochondrial gene set (Fig. 7). We found all the major clades within the phylogenetic tree had good supported values (BPP ≥0.97; BS ≥ 98). The 76 Basidiomycota species could be divided into 14 major clades in the phylogenetic tree, corresponding to the orders Agaricales, Boletales, Russulales, Polyporales, Hymenochaetales, Cantharellales, Pucciniales, Tremellales, Trichosporonales, Microbotryales, Sporidiobolales, Microstromatales, Ustilaginales, and Tilletiales (Table S8). The 22 Agaricales species could be divided into four major groups, wherein the first comprised two species, *C. fumosa* and *Hygrophorus russula* (Li et al. 2019d), the second group comprised four species from the *Pleurotus* genus (Li et al. 2018a). The results indicated that the *C. fumosa* mitogenome exhibited a close evolutionary relationship with *Hygrophorus russula*.

## Discussion

### Size expansion of the *C. fumosa* mitogenome

In this study, we obtained the complete mitogenome of *C. fumosa*. The 256,807 bp mitogenome of *C. fumosa* was the second largest among all reported fungal mitogenomes, which was only lower than that of *Morchella importuna* (272,238 bp) (Liu et al. 2019) from the phylum Ascomycota. The mitogenome of *C. fumosa* was the largest among all Basidiomycota mitogenomes reported, which was 20.96 kb larger than the previously reported largest mitogenome of Basidiomycota, *Rhizoctonia solani* (235,849 bp) (Losada et al. 2014). Several factors were considered closely related to the expansion of the *C. fumosa* mitogenome. Firstly, the mitogenome of *C. fumosa* contained a relatively loose structure (Ye et al. 2020), which was considered to be the main factors leading to the size expansion of the *C. fumosa* mitogenome. In addition, the *C. fumosa* mitogenome contained the largest number of introns and intronic ORFs among all fungal mitogenomes published, and the large number of introns and intronic ORFs was also one of the main factors leading to the increase of the size of *C. fumosa* mitogenome. A total of 38.23% of repeat sequences and 16 plasmid-derived genes (DNA polymerases and RNA polymerases) were detected in the *C. fumosa* mitogenome, which also promoted the expansion of the *C. fumosa* mitogenome. In conclusion, large intergenic regions, intronic regions, accumulation of repeat sequences and plasmid-derived genes together promoted the *C. fumosa* mitogenome become the largest mitogenome among Basidiomycota species.

### Gene rearrangements in Agaricales species

The arrangement of mitochondrial genes could provide reference information for understanding the origin and evolution of fungal species (Beaudet et al. 2013; Sankoff et al. 1992). Among the 22 Agaricales species we tested, only two species from the *Moniliophtora* genus and two species from the *Pleurotus* genus had identical gene arrangements, indicating their close phylogenetic relationships. However, large-scale gene rearrangements were observed in species from different genera, even within the same genera, indicating that the mitochondrial gene arrangement of Agaricales species had high variability. Gene arrangement analysis indicated that gene shifts and inversions occurred in the mitogenome of *C. fumosa*, which made the *C. fumosa* mitogenome have a unique gene arrangement in Agaricales species. Mitochondrial gene rearrangement has been widely studied in animals, and several models have been proposed to reveal the mechanism of gene rearrangement in animals, such as the tandem duplication-random loss (TDLR) (Xia et al. 2016) and duplication and nonrandom loss model (Lavrov et al. 2002). Compared with animals, fungal gene arrangements have larger variations and rearrangements occur on a larger scale (Aguileta et al. 2014). Gene rearrangements in fungi have been less studied, and the mechanism of mitochondrial gene rearrangement in fungi was still unknown. The mechanism of large-scale rearrangement in Agaricales species needs to be further studied to understand the evolution of fungi.

### Variations of gene content in Agaricales mitogenomes

According to the second parity rule, as long as there is no mutation or selection bias, each base in the complementary DNA strand exists at approximately equal frequencies (Chen et al. 2014). However, frequent GC skews and AT skews were observed in mitogenomes of Agaricales species. The presence of AT or GC skews on the same DNA strand from different species indicated that mitogenomes of different species underwent different mutations or environmental selection. In addition, we observed that the potential positive selection of *nad3* and *rps3* genes occurred between different Agaricales species. Different Agaricales species have different growth media, different environmental conditions and different life styles. The results showed that the two genes may have been subjected to strong pressure of positive selection in the process of environmental adaptation of different mushrooms.

### Intron dynamics of Agaricales mitogenomes

Most of the Basidiomycota introns were found belonged to the group I, while *cox1* gene was considered as the largest host gene of group I introns. On average, more than 40% of fungal introns were found harboring in the *cox1* gene. In this study, the dynamic changes of introns in the *cox1* gene of 22 Agaricales species were analyzed, and we found that intron gain/loss events occurred in the mitogenome of Agaricales. In addition, Pcl K and AI were found to be the most common homologous introns in the order Agaricales, while Pcls B, H, U, Z, and AG only existed in one of the Agaricales 22 species. The rare introns in the order Agarciales have been detected in some distant species, such as *Rhizopogon vinicolor* (Li et al. 2019a) and *Rhizophydium* sp. 136 (Férandon et al. 2010), indicating potential horizontal gene transfer events. The *cox1* gene of *C. fumosa* contained the largest number of introns among the 22 Agaricales species, while four novel Pcls were detected in the *cox1* gene of *C. fumosa*. Intron considered as an important genetic element in the mitogenome of fungi (Burke 1988; Gomes et al. 2018), and its dynamic changes can significantly affect the organization and size of the mitogenome of fungi (Hamari et al. 2002; Himmelstrand et al. 2014). The origin and evolution of the novel Pcls needed to be further analyzed to understand the evolution of the *C. fumosa* mitogenome.

### Phylogenetic analysis of Basidiomycota

Just like *C. fumosa*, many Basidiomycota species have few morphological characteristics, and some of morphological characteristics are easy to overlap, which makes it difficult to classify species accurately only according to morphology. Mitochondrial core PCGs and rRNA genes were considered as a powerful tool to analyze the phylogeny of species because of its unique advantages (Paquin et al. 1997; Wang et al. 2018). As more and more mitogenomes of Basidiomycota are sequenced, it will be helpful to reveal the phylogenetic relationships of Basidiomycota species. In this study, we obtained a phylogenetic tree of 76 Basidiomycota species with high support value based on the combined mitochondrial gene set. More than two-thirds of Basidiomycota mitogenomes available in the NCBI database were included for phylogenetic analysis in this study. The results showed that mitochondrial core PCGs and rRNA genes were effective molecular markers to analyze the phylogenetic relationships of Basidiomycota. More mitogenomes of Basidiomycota are needed for reconstructing evolution of Basidiomycota.

## Conclusion

In this study, the mitogenome of *C. fumosa* was sequenced, assembled and compared. The mitogenome of *C. fumosa* was the largest among all Basidiomycota mitogenomes reported. Large intergenic regions, intronic regions, accumulation of repeat sequences and plasmid-derived genes together promoted the size expansion of the *C. fumosa* mitogenome. Comparative mitogenome analysis indicated the number of PCGs, base compositions, gene contents and tRNA genes varied between the 22 Agaricales mitogenomes tested. In addition, we found that the *rps3* gene was subjected to strong pressure of positive selection between some Agaricales species. The *C. fumosa* mitogenome had a unique gene order among Agaricales species, and large-scale gene rearrangements occurred between Agaricales species. Intron loss/gain and horizontal gene transfer events occurred in introns of *cox1* genes in Agaricales, which promoted the organization and size variations of Agaricales mitogenomes. Phylogenetic analysis indicated that mitochondrial genes were reliable molecular markers to analyze the phylogenetic relationships of Basidiomycota. This study served as the first report on the mitogenome from the family Clavariaceae, which will promote the understanding of the genetics, evolution and taxonomy of *C. fumosa* and related species.

## Supplementary Information

**Additional file 1: Figure S1.** Putative secondary structures of tRNA genes in the mitochondrial genome of *Clavaria fumosa*. All genes are shown in order of occurrence in the mitogenome of *C. fumosa*, starting from *trnF*.

**Additional file 2: Table S1.** Comparison on mitochondrial genomes among 22 Agaricales species. **Table S2.** Characterization of the mitochondrial genome of *Clavaria fumosa*. **Table S3.** Start and stop codon analyses of 15 core protein-coding genes in 22 Agaricales species. **Table S4.** Local BLAST analysis of the *Clavaria fumosa* mitochondrial against itself. **Table S5.** Tandem repeats detected in the mitochondrial genome of *Clavaria fumosa* using the online program Tandem Repeats Finder. **Table S6.** Distribution of repeat loci in the mitochondrial genome of *Clavaria fumosa* searched by REPuter. **Table S7.** Local BLAST searches between the mitochondrialand the nuclear genomes of *Clavaria fumosa*. **Table S8.** Species, and GenBank accession number used for phylogenetic analysis in this study.

## Data Availability

All data generated or analyzed during this study are included in this published article [and its supplementary information files].

## Abbreviations

mitogenome: Mitochondrial genome
BI: Bayesian inference
ML: Maximum likelihood
PCG: Protein-coding gene
Pcls: Position classes
*Ks*: Synonymous substitution rates
*Ka*: Nonsynonymous substitution rates
